# Novel RF Interrogation of a Fiber Bragg Grating Sensor Using Bidirectional Modulation of a Mach-Zehnder Electro-Optical Modulator

**DOI:** 10.3390/s130708403

**Published:** 2013-07-02

**Authors:** Sang-Jin Choi, Wankai Mao, Jae-Kyung Pan

**Affiliations:** 1 Department of Electrical Engineering and Smart Grid Research Center, Chonbuk National University, Jeonbuk 561-756, Korea; E-Mail: sang_jin@jbnu.ac.kr; 2 Samsung Techwin Opto-Electronics Corporation, 230 Tianhe Road, Tianhe District, Guangzhou 510620, China; E-Mail: wankai.mao@samsung.com

**Keywords:** radio-frequency (RF) interrogation, fiber Bragg grating (FBG), chirped fiber Bragg grating (CFBG), Mach-Zehnder electro-optical modulator (MZ-EOM)

## Abstract

We propose and experimentally demonstrate the novel radio-frequency (RF) interrogation of a fiber Bragg grating (FBG) sensor using bidirectional modulation of a Mach-Zehnder electro-optical modulator (MZ-EOM). Based on the microwave photonic technique and active detection, the transfer function of the proposed system was obtained, and the time delay was calculated from the change in the free spectral range (FSR) at different wavelengths over the optimal measuring range. The results show that the time delay and the wavelength variation have a good linear relationship, with a gradient of 9.31 ps/nm. An actual measurement taken with a sensing FBG for temperature variation shows the relationship with a gradient of 0.93 ps/10 °C. The developed system could be used for FBG temperature or strain sensing and other multiplexed sensor applications.

## 1. Introduction

The use of fiber Bragg gratings (FBGs) in sensor applications has been extensively studied for the past 20 years. FBGs are lightweight, offer low power consumption and multiplexing capability, are resistant to electromagnetic wave interference, and offer high sensitivity to strain and temperature. FBG sensors, because of their small size, are ideal for the health monitoring of smart structures since they can easily be embedded inside structural members [1]. Several FBG sensing interrogation methods, which can be classified into passive detection and active detection, have been reported [2], and successful schemes include the use of scanning Fabry-Perot or acoustic filters, tuning lasers, and charge coupled device (CCD) spectrometers. Each of these approaches has its own advantages, and commercial systems that make use of these techniques are now available.

Recently, FBG sensor interrogation systems employing a Mach-Zehnder electro-optical modulator (MZ-EOM) have gained the attention of researchers due to the capability of the MZ-EOM in handling fast signal processing speeds. By adopting fiber Sagnac-loop-based microwave photonic filtering, the high-frequency wavelength variation of a sensing FBG can be converted into the intensity change of the recovered RF signal [3]. Through the use of a dispersion compensation fiber, an FBG interrogator can convert the sensing wavelength variation measurement to a time-domain measurement at a speed on the order of megasamples per second [4]. Bidirectional modulation of MZ-EOMs has been used to obtain optical fiber chromatic dispersion measurements [5]. In this paper, we propose and demonstrate the RF interrogation of an FBG sensor using the bidirectional modulation of an MZ-EOM.

## 2. Measurement Method

**Figure 1.**
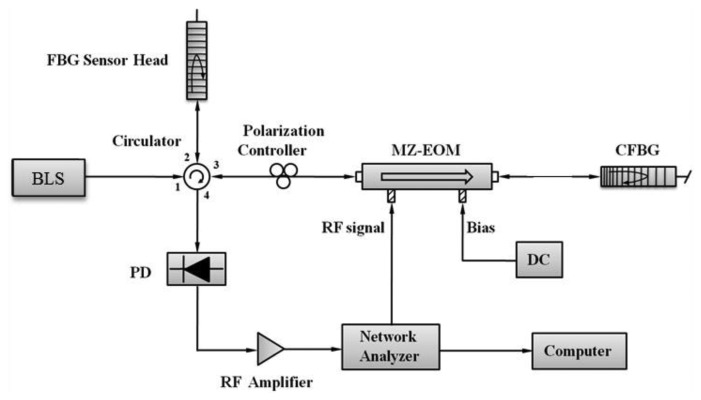
Setup for the proposed RF interrogation structure of an FBG sensor using bidirectional modulation of an MZ-EOM. BLS: broadband light source. MZ-EOM: Mach-Zehnder electro-optical modulator. CFBG: chirped fiber Bragg grating. PD: photodetector.

The proposed RF interrogation structure of an FBG sensor, which consists mainly of a broadband light source (BLS), FBG, MZ-EOM, chirped fiber Bragg grating (CFBG), and photodetector (PD), is shown in Figure 1.

**Figure 2.**
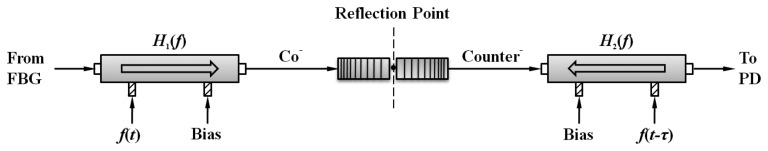
Equivalent model of bidirectional modulation with a MZ-EOM and a CFBG.

Light from the BLS passes through port 1 of a four-port circulator and is reflected by the FBG sensor head in port 2. After passing through port 3 and polarization controller, the light is modulated by an RF signal via the MZ-EOM with co-propagation, *i.e.*, propagation in the same direction as the light (as indicated by the arrow in the diagram of the MZ-EOM shown in Figure 1). The unidirectional modulated light is reflected by the CFBG and is modulated by an RF signal via the MZ-EOM with counter-propagation, *i.e.*, propagation in the opposite direction to the light. The bidirectional modulated light is received by the PD and measured by the network analyzer (NA). The modulating process in Figure 1 can be considered as an equivalent model, shown in Figure 2, with two MZ-EOMs cascaded in series [5].

The travel time in terms of the wavelength of the proposed structure, *τ*(*λ*), can be expressed as:

(1) $$\begin{array}{c} \tau (\lambda )=\tau_{\text{fiber}}+\tau_{\text{CFBG}}(\lambda )=\frac{2L_{\text{fiber}}}{v_{g}}+\frac{(\lambda_{0}-\lambda )\cdot 2L_{\text{CFBG}}}{\Delta \lambda_{\text{chirp}}\cdot v_{g}}\\ \left(\text{for}\;2n_{\text{eff}}\;\Lambda_{\text{short}}<\lambda <2n_{\text{eff}}\Lambda_{\text{long}}\right) \end{array}$$

where *τ_fiber_* is the travel time spent in the optical fiber between the MZ-EOM and CFBG, is the travel time spent in the CFBG [6], *L_fiber_* is the length of the optical fiber between the MZ-EOM and CFBG, *L_CFBG_* is the grating length of the CFBG, *v_g_* is the group velocity of light in the optical fiber, *λ*_0_ is the central wavelength of the CFBG, Δ*λ_chirp_* is the chirped bandwidth of the CFBG, *n_eff_* is the effective index of refraction of the CFBG, and *Λ_short_* and *Λ_long_* are the shortest and longest periods in the CFBG, respectively. In Equation (1), the wavelength of the FBG sensor head has to be in the range of the chirped bandwidth of the CFBG, which is determined by the shortest and longest periods in the CFBG.

The time delay, Δ*_τ_*, which is the difference in the travel time for two wavelengths (*λ*_1_, *λ*_2_), can be expressed as:

(2) $$\Delta \tau =\tau (\lambda_{2})-\tau (\lambda_{1})=\frac{2L_{\text{CFBG}}\cdot \Delta \lambda_{\text{FBG}}}{\Delta \lambda_{\text{chirp}}\cdot v_{g}}$$

where Δ*λ_FBG_* is the wavelength variation of the FBG sensor head in Figure 1. From Equation (2), we can see that the narrower chirped bandwidth of the CFBG with a longer grating length increases the accuracy of the proposed system. In addition, the time delay corresponding to the wavelength variation of the FBG sensor head can be determined for a given CFBG.

In the course of propagation, the first MZ-EOM in Figure 2 causes the forward propagating light in Figure 1 to experience co-propagating modulation by the RF signal *f*(*t*). On the other hand, the second MZ-EOM in Figure 2 causes the back-reflected light in Figure 1 to experience counter-propagating modulation caused by the RF signal *f*(*t-τ*). Assuming that the modulation indices are very small and the biasing is set at the quadrature point, we can obtain an expression for the output optical power *P_out_*(t) at the PD as [5]:

(3) $$p_{\text{out}}(t)=\frac{P_{\text{in}}T_{D}}{4}[1+m_{1}H_{1}(f)\cos2\pi ft]\cdot [1+m_{2}H_{2}(f)\cos2\pi f(t-\tau )]$$

where *P_in_* is the input optical power, *T_D_* indicates the coupling and optical transmission losses of the structure, *m*_1_ and *m*_2_ are the modulation indices of co-propagating and counter-propagating modulation, respectively, and *H*_1_(*f*) and *H*_2_(*f*) are the transfer functions for co-propagating and counter-propagating modulation of the MZ-EOM, respectively.

The DC and harmonic components in Equation (3) are eliminated at the PD and the NA because all vector NAs use a tuned-receiver (narrow-band) architecture to reject harmonic and spurious signals. Consequently, if it is assumed that *m*_1_=*m*_2_=*m*, the total transfer function *H*(*f*) measured at the NA can be written as:

(4) H(f)=A0[H1(f)+e−j2πfτH(f)2]

where *A*_0_=*mRG_m_P_in_T_D_*/4, is the responsivity of the PD, and *G_m_* is the gain of the RF amplifier. The free spectral range (FSR) is formed by ripples in the transfer function and depends on the travel time in Equation (4), which is in turn strongly related to the period of the ripples [7]. For each wavelength, the FSR can be expressed as:

(5) $${\text{FSR}}_{\lambda }=\frac{1}{\tau (\lambda )}$$

Upon measuring the change in the FSR based on the wavelength variation, the travel time and time delay can be calculated from Equations (1,2,5).

## 3. Experiments and Discussion

For the experimental setup shown in Figure 1, we used a BLS with a power of -1.5 dBm/nm around 1,550 nm (LiComm OFB-BCM-21AP), an FBG sensor head with a central wavelength of 1,549.96 nm, an MZ-EOM with a bandwidth of 10 GHz (Photoline MXAN-LN-10), a CFBG with a central wavelength of 1,550.48 nm, a chirped bandwidth of 2.88 nm, a grating length of 27.4 mm, a PD with a bandwidth of 25 GHz (New Focus Model 1414), an RF amplifier with a gain of 25 dB (Mini-Circuits ZHL-6A), and an NA with an output RF signal of 10 dBm and 1601 sampling points (Agilent E5061B). The DC bias voltage was set at 2.50 V. Due to the polarization effect, one polarization controller had to be added in front of the MZ-EOM, and the optical fiber length between the MZ-EOM and CFBG was 2.562 m.

**Figure 3.**
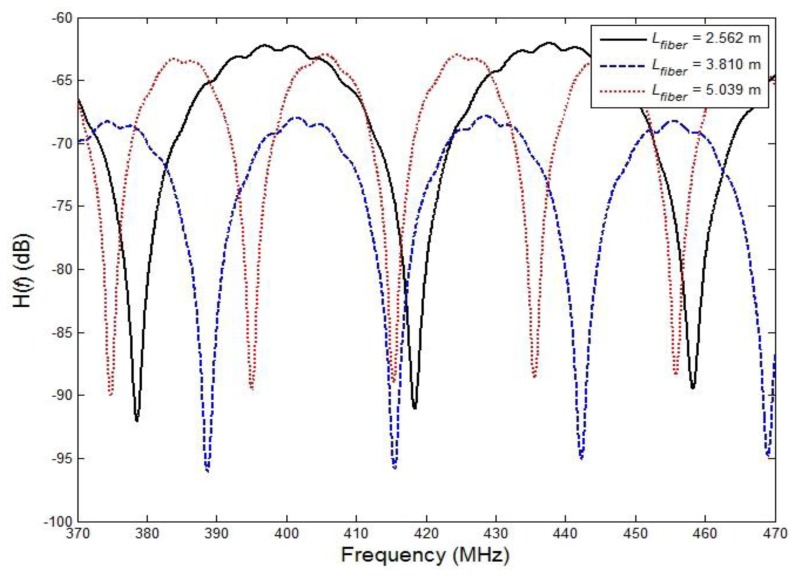
Measured transfer function *H*(*f*) for three different optical fiber lengths between an MZ-EOM and CFBG: *L_fiber_*= 2.562 m, *L_fiber_*= 3.810 m, and *L_fiber_*= 5.039 m.

Figure 3 shows the experimental results obtained with the proposed RF interrogation system, namely the transfer function *H*(*f*) for three different optical fiber lengths between the MZ-EOM and CFBG over a modulation frequency range of 370 MHz to 470 MHz. From the peak values, it can be determined that the FSRs in Equation (5) and the travel times in Equation (1) for *L_fiber_*= 2.562 m, 3.810 m, and 5.039 m are 39.853238 MHz and 25.09206 ns, 26.803879 MHz and 37.30803 ns, and 20.260937 MHz and 49.35606 ns, respectively. Obviously, as the optical fiber length increases, the FSR is reduced and the travel time increases. To distinguish the wavelength variation, it is very helpful if the optical fiber length can be as short as possible. Furthermore, the characteristics of the two transfer functions, *H*_1_(*f*) and *H*_2_(*f*), should be quite different, except when the modulation frequency range is significantly lower than the bandwidth of the MZ-EOM, in which case the optimal measuring range should be selected near a frequency of 500 MHz.

**Figure 4.**
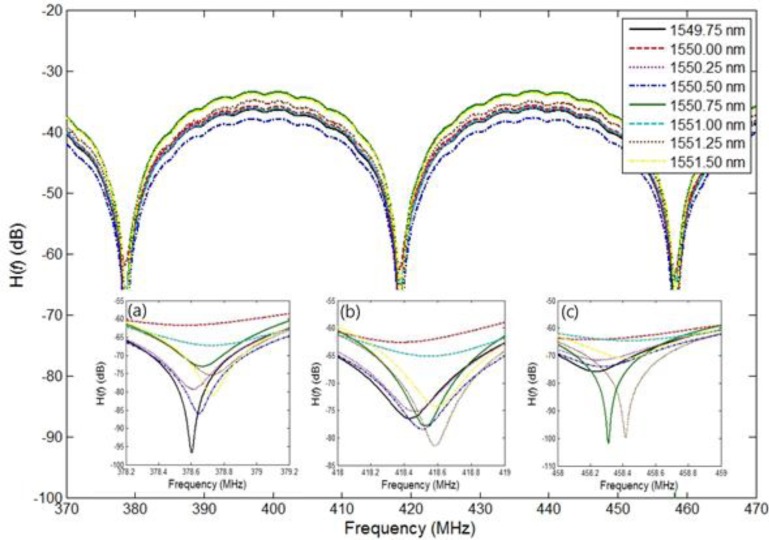
Measured transfer function *H*(*f*) for eight wavelengths (from 1,549.75 nm to 1,551.50 nm in 0.25 nm steps) with *L_fiber_* 2.562 m and a tunable laser wavelength of 1549.6 ∼ 1551.6 nm in the frequency range of 370 MHz to 470 MHz, (**a**) *H*(*f*) in the frequency range of 378.2 MHz to 379.2 MHz; (**b**) *H*(*f*) in the frequency range of 418.0 MHz to 419.0 MHz; (**c**) *H*(*f*) in the frequency range of 458.0 MHz to 459.0 MHz.

**Figure 5.**
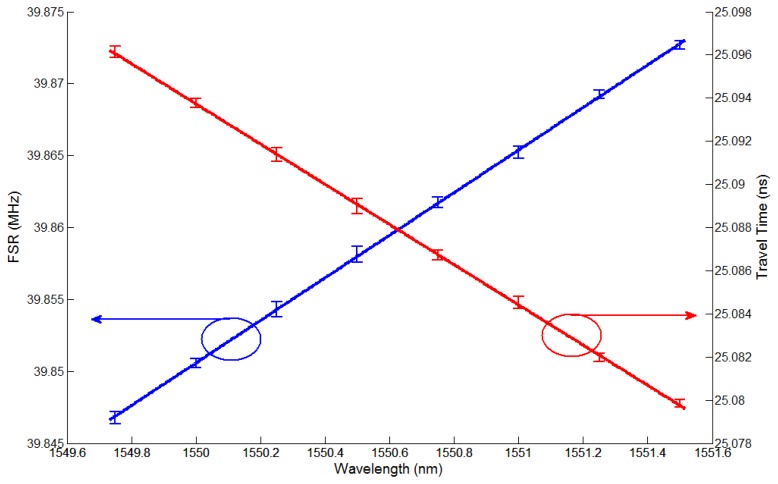
The FSR (blue line) and the travel time (red line) according to wavelength variation of the laser source.

An additional experiment was conducted to investigate the issue of wavelength variation resolution. For convenience, we used a tunable laser source instead of the wavelength variation of the FBG sensor head. Figure 4 shows the experimental results obtained for the transfer function *H*(*f*) for eight wavelengths (ranging from 1,549.75 nm to 1,551.50 nm in 0.25 nm steps) in the frequency range of 370 MHz to 470 MHz when a tunable laser source was used. To read a more accurate value, we chose three frequency ranges in a 1 MHz span, thus 378.2 MHz to 379.2 MHz, 418 MHz to 419 MHz, and 458 MHz to 459 MHz as shown in Figure 4(a–c). Since the NA has 1601 sampling points, the measurement resolution of a 1 MHz span will be 0.625 kHz. Furthermore Figure 4(a–c) show the values with a mean of 100 measured values and a smoothing effect of 20%. From these values, we can determine the FSRs for the eight wavelengths shown in Figure 4, which are shown in Figure 5. Also, Figure 5 shows travel times corresponding to the FSRs for the eight wavelengths, which are 25.09606 ns and 25.07976 ns at the first wavelength of 1,549.75 nm and last wavelength of 1,551.50 nm, respectively. We measured each value five times for each of the different wavelengths and plotted two straight lines using the linear polynomial type fitting with least squares method. In all cases, relative errors were within 3%. We note that the relationship between the time delay and the wavelength variation is linear with a gradient of 9.31 ps/nm.

**Figure 6.**
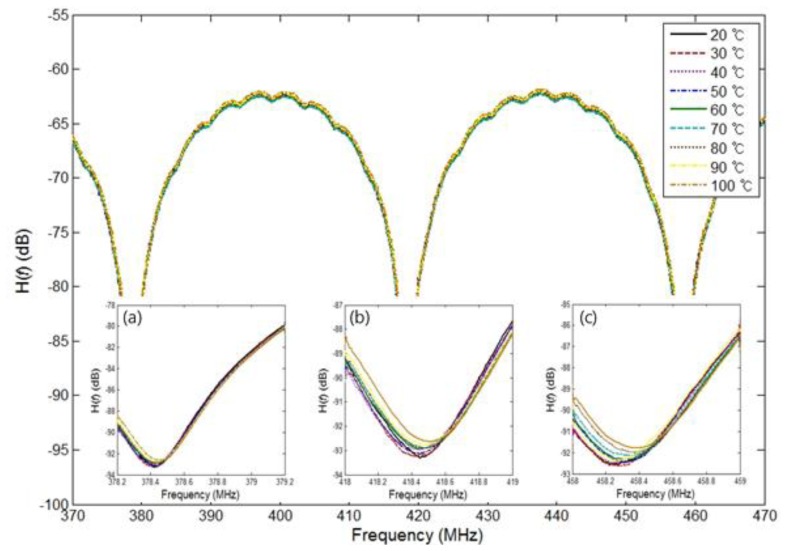
Measured transfer function *H*(*f*) for nine temperatures (from 20 °C to 100 °C in 10 °C steps) with *L_fiber_* 2.562 m in the frequency range of 370 MHz to 470 MHz, (**a**) *H*(*f*) in the frequency range of 378.2 MHz to 379.2 MHz; (**b**) *H*(*f*) in the frequency range of 418.0 MHz to 419.0 MHz; (**c**) *H*(*f*) in the frequency range of 458.0 MHz to 459.0 MHz.

To apply the proposed structure with an actual measurement, we measured the temperature with an FBG sensor head with a central wavelength of 1,549.96 nm, an FWHM of 0.47 nm, and a reflectance of 77.664%. An FBG sensor head is put in a temperature chamber to help control the temperature. Figure 6 shows the experimental results of the transfer function *H*(*f*) as a function of the temperature at 20 °C to 100 °C in 10 °C steps in the frequency range of 370 MHz to 470 MHz. Also, we chose three frequency ranges in a 1 MHz span, 378.2 MHz to 379.2 MHz, 418 MHz to 419 MHz, and 458 MHz to 459 MHz, as shown in Figures 6(a–c), which show the values with a mean of 100 measured values and a smoothing effect of 20%.

**Figure 7.**
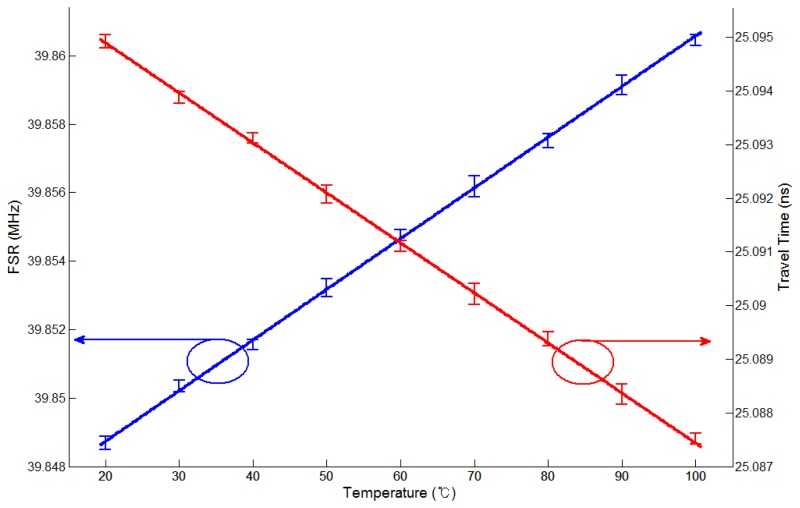
The FSR (blue line) and the travel time (red line) according to the temperature variation of the FBG sensor head.

From these values, the FSRs are determined for nine temperatures, which are shown in Figure 7. Figure 7 also shows travel times corresponding to the FSRs for nine temperatures, which are 25.09490 ns and 25.08744 ns at the first temperature of 20 °C and the last temperature of 100 °C, respectively. We confirmed that the relationship between the time delay and the temperature variation is linear with a gradient of 0.93 ps/10 °C. The relative errors in Figure 7 are larger than those in Figure 5 due to the inaccuracy of the temperature chamber. The system performance can be further improved by using a shorter optical fiber length between MZ-EOM and CFBG, a longer grating length of the CFBG, an increased number of NA sampling points, and an optical source with larger output power.

## 4. Conclusions

We have proposed and experimentally demonstrated the RF interrogation of an FBG sensor using bidirectional modulation of an MZ-EOM. The transfer functions for wavelength variation and temperature variation of the proposed system were obtained experimentally. The calculated travel time from the transfer function results in a time delay for the sensing wavelengths over the optimal measuring range. In this study, a good linear relationship between the time delay and wavelength variation with a gradient of 9.31 ps/nm was achieved. An actual measurement for temperature variation with the proposed structure shows that the time delay and the temperature variation have a good linear relationship with a gradient of 0.93 ps/10 °C. Therefore, the proposed FBG sensor RF interrogation system shows potential for use in FBG temperature or strain sensing and other multiplexed sensor applications.
